# Evaluation of Bone and Soft Tissue Tumors of the Shoulder Girdle

**DOI:** 10.7759/cureus.46162

**Published:** 2023-09-28

**Authors:** İsmail Büyükceran, Şafak Aydın Şimşek, Ercan Bayar, Tolgahan Cengiz, Hüseyin Sina Coşkun, Nevzat Dabak

**Affiliations:** 1 Department of Orthopedics and Traumatology, Ondokuz Mayis University, Faculty of Medicine, Samsun, TUR; 2 Department of Orthopedics and Traumatology, Inebolu State Hospital, Kastamonu, TUR

**Keywords:** bone tumors, soft tissue neoplasms, bone neoplasms, shoulder girdle, shoulder

## Abstract

Introduction: The shoulder girdle comprises the scapula, clavicle, proximal humerus, and the soft tissues surrounding these structures. Bone and soft tissue tumors are notably more prevalent in the lower extremity than in the upper extremity. However, the shoulder ranks as the third most common site for primary tumors, following the hip-pelvis and knee.

Materials and methods: This study conducted a retrospective examination of patients who presented with pain and swelling in the shoulder and its vicinity. The evaluation was carried out using a multidisciplinary approach by the Bone and Soft Tissue Tumors Council.

Results: The study included 224 patients diagnosed with a tumoral lesion in the shoulder girdle between 2004 and 2021. Among these patients, 22 were assessed to have lesions other than tumors, while 105 (51.98%) had benign lesions, and 97 had malignant lesions. The most prevalent benign lesions were cystic bone lesions (30) and soft tissue lipomas (10). The primary form of malignant lesion was metastatic tumors (49).

Conclusion: Pathologies in the shoulder girdle may manifest through pain, palpable swelling, pathological fractures, or may be incidentally detected via radiological imaging. Notably, pain, hypercalcemia, and pathological fractures are significant indicators, especially in cases of bone metastases, which often follow a highly fatal course when involving long bones. The musculoskeletal system is the third most common site for metastasis, following the lungs and liver. Hence, particular attention should be directed toward metastatic concerns in the shoulder and its surrounding area.

## Introduction

The shoulder girdle comprises the scapula, clavicle, proximal humerus, and the soft tissues surrounding these structures. The incidence of bone and soft tissue tumors in the upper extremities is one-third that of the lower extremities. The shoulder is where soft tissue tumors are most often seen, following the hip-pelvis and knee [1-2]. Although bone tumors can be seen in all bone structures, bone tumors in the shoulder region are most often seen in the proximal humerus, the fourth most common bone localization of primary bone tumors [3-4]. It has been reported that 10-15% of osteosarcomas and 10% of Ewing sarcomas are seen in the proximal humerus [5-6].

the shoulder girdle ranks third in terms of soft tissue sarcoma localization. The most common localizations of sarcomas within the shoulder girdle are the deltoid muscle and fossa supraspinatus [1-2]. The shoulder girdle is one of the most frequently seen localizations of metastatic tumors. Although managing pain and regaining function are the main goals in treating metastases, palliative measures are frequently used. In cases involving metastases and lytic lesions with a fracture or a high risk of fracture, surgical intervention is planned, considering the patient's life expectancy and associated health issues [7-8].

This study aimed to examine the distribution of patients with tumoral lesions around the shoulder who presented at the Orthopedics and Traumatology Clinic and were evaluated by the Bone and Soft Tissue Tumors Council and to increase awareness regarding the importance of considering tumoral lesions in cases presenting with discomfort in the shoulder region.

## Materials and methods

All patient evaluation records evaluated by the Bone and Soft Tissue Tumors Council between January 1, 2004, and January 1, 2021, were examined retrospectively. The Council consists of orthopedic oncologists, nuclear medicine specialists, radiology specialists, pathology specialists, oncologists, and pediatric hematology and oncology specialists. It is a community where patients with complex bone and soft tissue tumors are evaluated using a multidisciplinary approach, where further investigations are requested, and where treatment and follow-up plans are created for the patients. After obtaining ethics committee approval, all patient data were collected from the electronic medical records and patient files (approval number B.30.2.ODM.0.20.08/211, approval date: 11.05.2023). All procedures were carried out in accordance with the ethical rules and the principles of the Declaration of Helsinki.

The study focused on patients experiencing pain and swelling in the shoulder area, whose cases were deliberated within the Council. The scapula, clavicle, proximal humerus, and soft tissues surrounding these were evaluated as the shoulder region. Patient age, gender, complaints, pre-diagnosis, definitive diagnosis, and the decision of the Council were obtained from the Council forms. The clinical findings, radiological images, and pathology results, if present, were evaluated. The multidisciplinary Council played a crucial role in determining the necessary tests and devising appropriate treatment plans based on their collective expertise. Radiological and, when required, pathological diagnoses were conducted for all patients with bone and soft tissue tumors. The patients were divided into six groups: benign soft tissue tumors, benign bone tumors, malignant soft tissue tumors, malignant bone tumors, metastatic tumors, and diseases other than tumors.

## Results

About 224 patients with complaints of shoulder pain and swelling were evaluated, 113 were males and 111 were females, with a mean age of 39.3 years. Of these 224 patients discussed within the Council, 22 (9.82%) were evaluated as having a disease other than a tumor; seven with complaints associated with a chronic infection, two with a rotator cuff tear, two with changes secondary to trauma, six with findings associated with osteoarthritis and degeneration, two with Paget’s disease, and three with bursitis.

Of the remaining 202 patients, the lesions were evaluated as benign tumors in 105 (51.98%), benign bone lesions in 81, and benign soft tissue lesions in 24. The benign bone lesions were classified as 30 cystic lesions, 16 enchondromas, nine aneurysmal bone cysts, seven chondroblastomas, four osteoid osteomas, two eosinophilic granulomas, and one fibrous dysplasia (Table 1).

**Table 1:** Distribution of benign bone tumors

Benign bone tumors	(n=81)	%
Cystic lesion	30	37.03
Enchondroma	16	19.75
Osteochondroma	12	14.81
Aneurysmal bone cyst	9	11.11
Chondroblastoma	7	8.64
Osteoid osteoma	4	4.93
Eosinophilic granuloma	2	2.46
Fibrous dysplasia	1	1.23

A treatment plan was created according to the patient's age, tumor localization, and clinical status. Cystic lesions with no clinical complaints, which did not constitute a risk of fracture or cause cortical destruction, were followed up conservatively. In cases other than these that required surgery, the tumor was completely removed and then curettage and grafting were performed.

The 24 patients' benign soft tissue tumors were divided into 10 lipomas, five elastofibromas, five synovial chondromatoses, two desmoid tumors, one nodular fasciitis, and one schwannoma (Table 2). Five patients with synovial chondromatosis underwent synovectomy and an arthrotomy. Tumor excision was performed on the remaining 19 patients.

**Table 2:** Distribution of benign soft tissue Tumors

Benign soft tissue tumors	(n=24)	%
Lipoma	10	41.66
Elastofibroma	5	20.83
Synovial chondromatosis	5	20.83
Desmoid tumor	2	8.33
Nodular fasciitis	1	4.16
Schwannoma	1	4.16

Malignant lesions were found in 97 patients, malignant bone tumors in 27, malignant soft tissue tumors in 21, and metastasis in 49. The 27 malignant bone tumors were divided into 10 Ewing sarcomas, five plasmacytomas, four multiple myelomas, four chondrosarcomas, two osteosarcomas, and two lymphomas (Table 3).

**Table 3:** Distribution of malignant bone tumors

Malignant bone tumors	(n=27)	%
Ewing sarcoma	10	37.03
Plasmocytoma	5	18.51
Multiple myeloma	4	14.81
Chondrosarcoma	4	14.81
Osteosarcoma	2	7.4
Lymphoma	2	7.4

The 21 malignant soft tissue tumors were divided into seven malignant mesenchymal tumors, five liposarcomas, five synovial sarcomas, three pleomorphic sarcomas, and one fibrosarcoma (Table 4).

**Table 4:** Distribution of malignant soft tissue tumors

Malignant soft tissue tumors	(n=21)	%
Malignant mesenchymal tumor	7	33.33
Liposarcoma	5	23.8
Synovial sarcoma	5	23.8
Pleomorphic sarcoma	3	14.28
Fibrosarcoma	1	4.76

Among the 60 patients with metastasis, 24 were females and 36 were males. Lung cancer metastasis was found in 15 patients (eight males and seven females), renal cell cancer metastasis in 11 (six males and five females), hematopoietic system-origin metastasis in 11 (seven males and four females), breast cancer metastasis in nine, colon cancer metastasis in four, squamous cell cancer metastasis in four, prostate cancer metastasis in three, ovarian cancer in one, testis cancer metastasis in one, and hepatocellular cancer metastasis in one (Table 5).

**Table 5:** Distribution of metastatic tumors

Metastatic cancers	(n=60)	%
Lung cancer	15	25
Renal cell cancer	11	18.34
Hematopoietic system	11	18.34
Breast cancer	9	15
Colon cancer	4	6.67
Squamous cell cancer	4	6.67
Prostate cancer	3	5
Ovarian cancer	1	1.66
Testis cancer	1	1.66
Hepatocellular cancer	1	1.66

**Figure 1:**
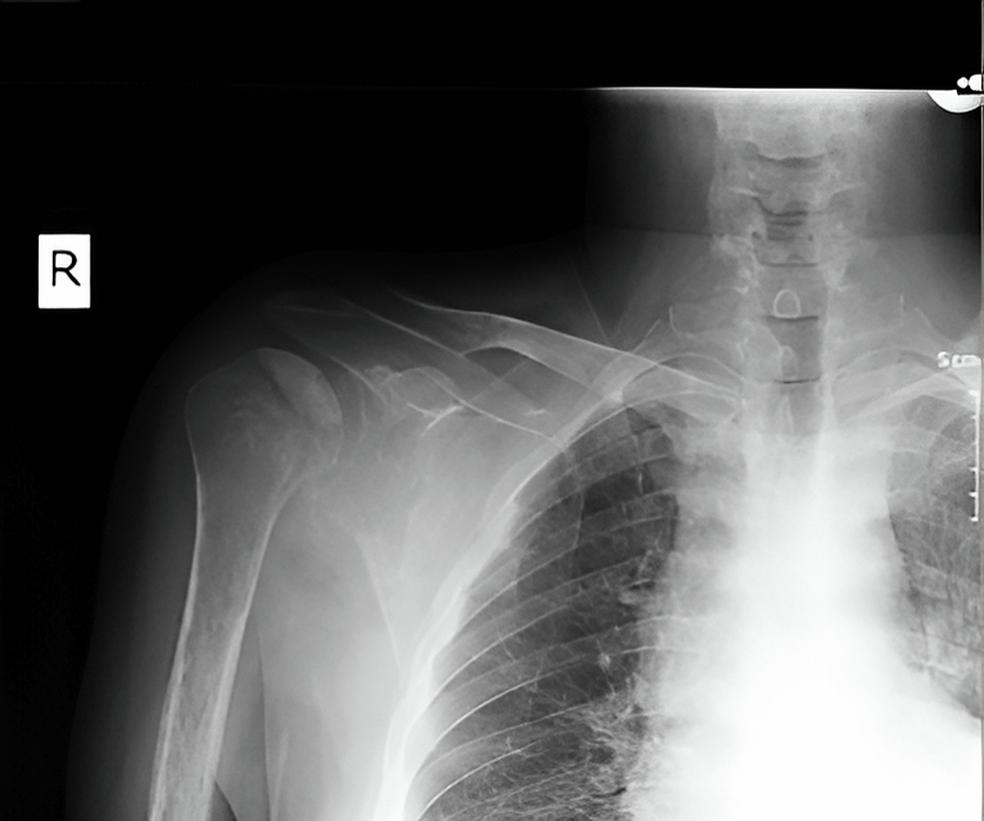
Preoperative X-ray image of the humerus head and scapula

**Figure 2:**
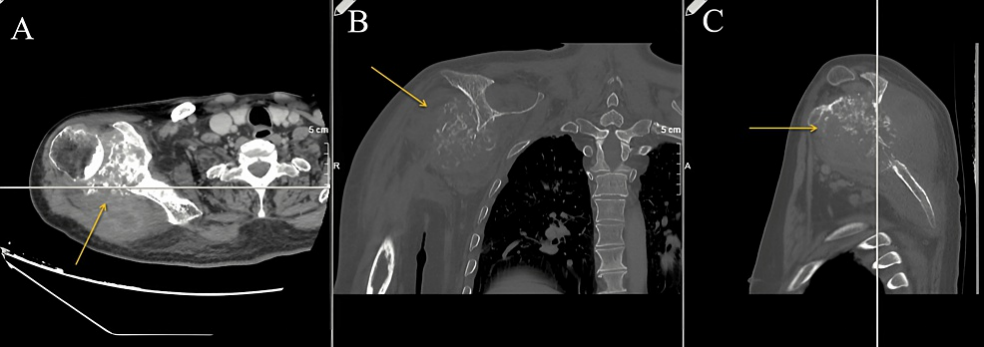
Preoperative axial (A), coronal (B), and sagittal (C) CT images of the shoulder. Lytic infiltrative lesions were observed extending from the scapula corpus to the glenoid and coracoid process and completely infiltrating the glenoid and sclerotic lesions in the part of the humerus head facing the glenoid

**Figure 3:**
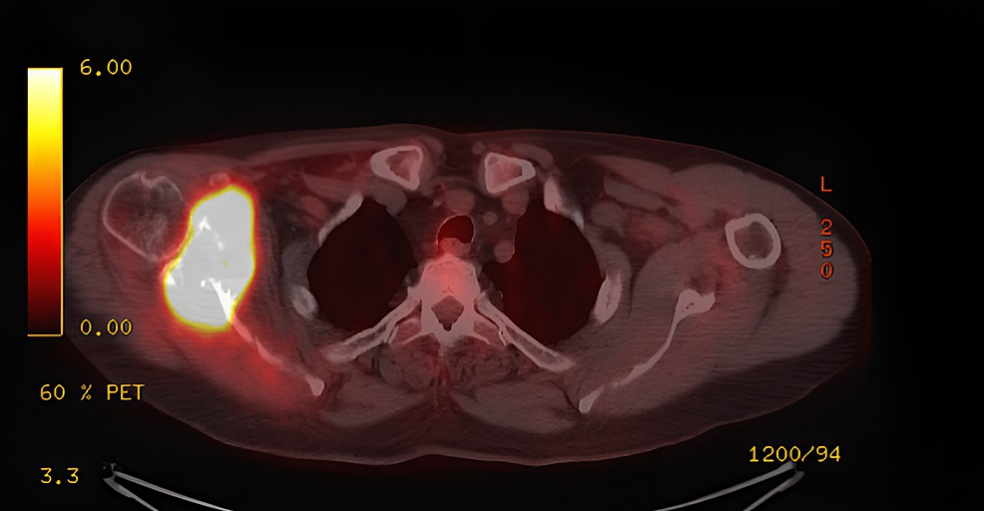
PET image of the metastasis in the scapula

**Figure 4:**
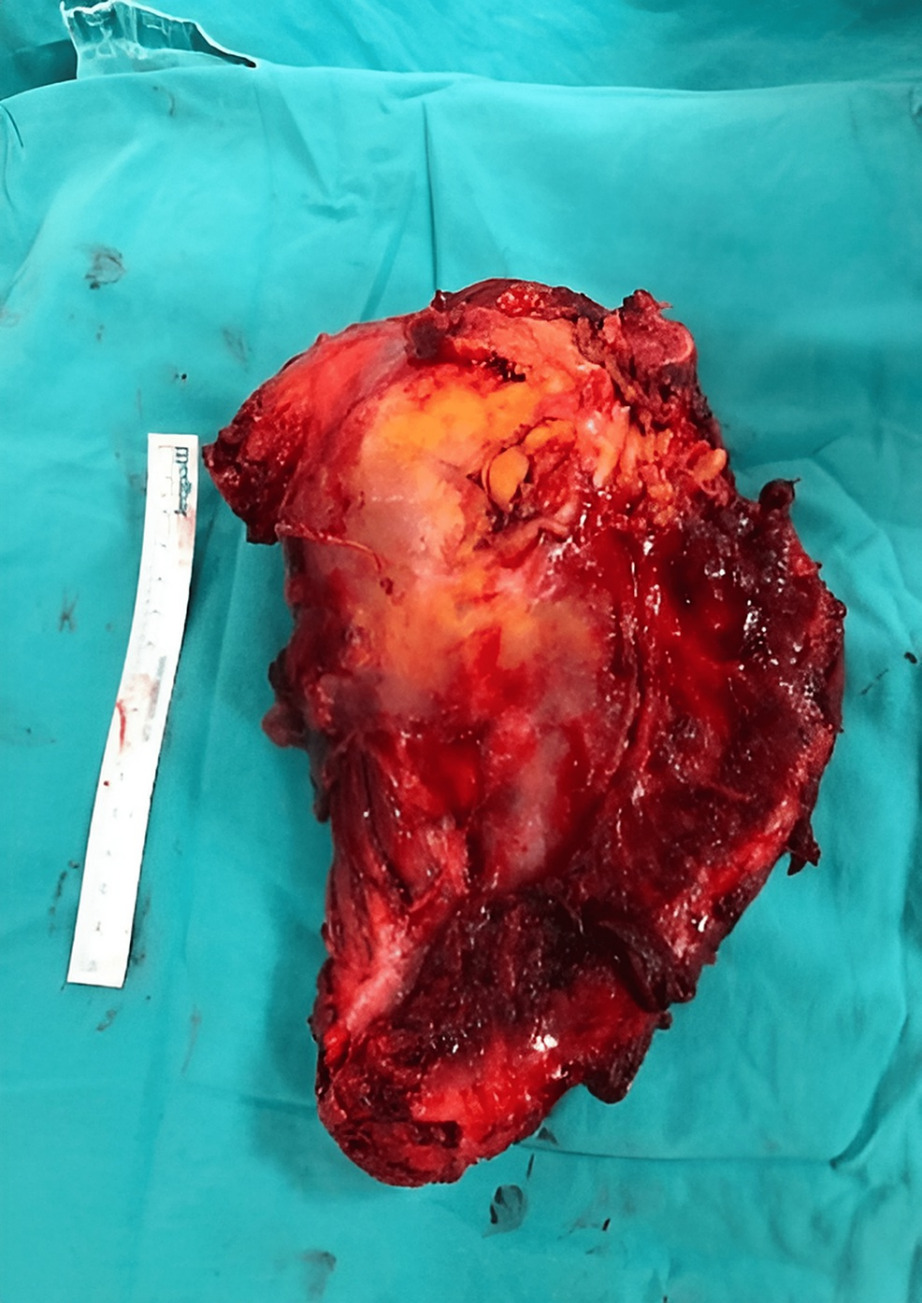
Image of the intraoperatively resected material

**Figure 5:**
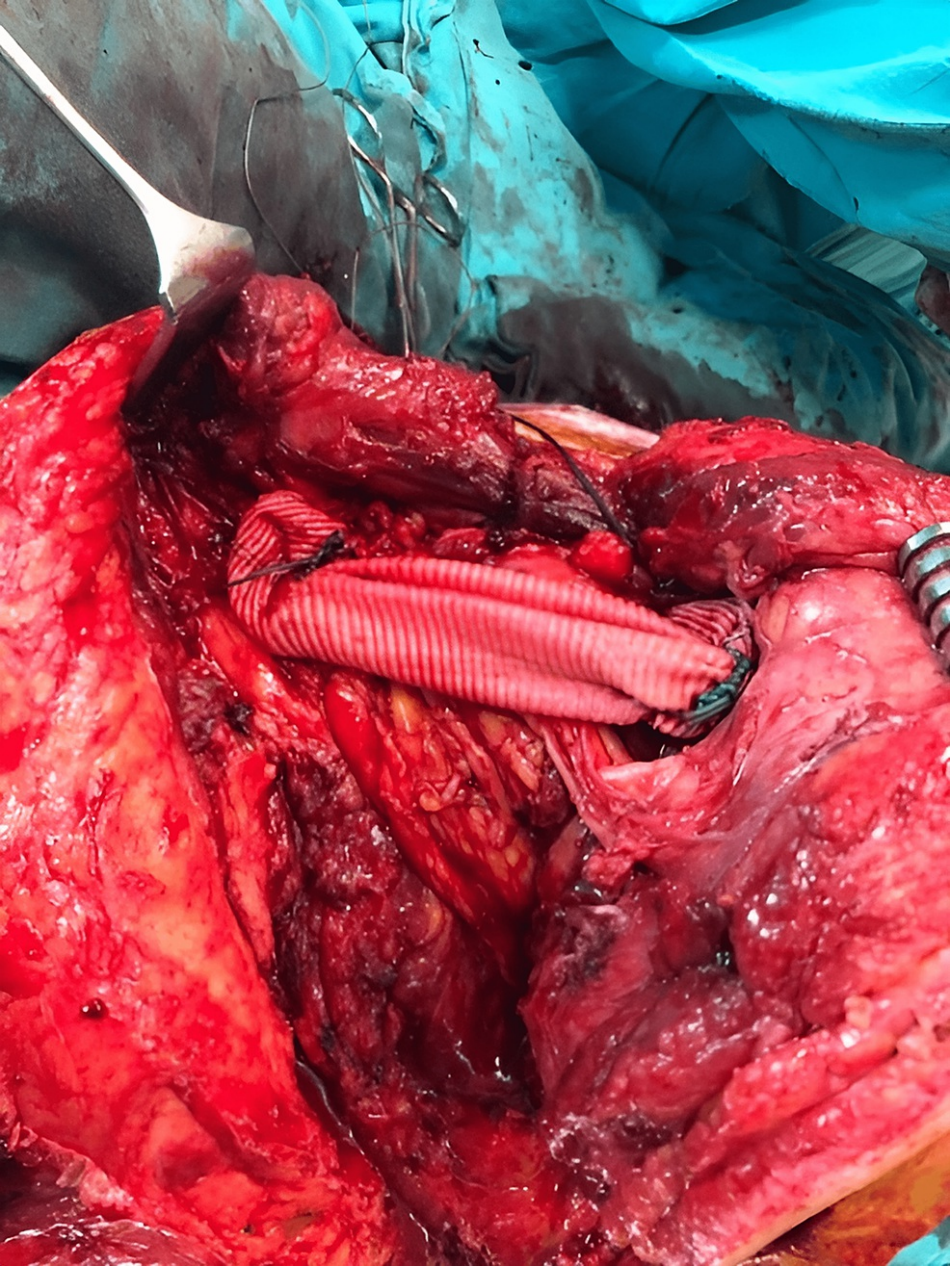
Intraoperative fixation of the upper extremity to the distal clavicle using an aortic graft

The following figures show an illustration of the patient who underwent surgery in our clinic as a result of colon adenocarcinoma scapula metastasis. A 52-year-old male had undergone a biopsy at another center because of complaints of newly developed numbness and weakness in his right arm. The biopsy results were consistent with metastasis, and in further tests, the patient was diagnosed with colon adenocarcinoma. X-ray imaging revealed extensive destruction in the scapula (Figure 1). On CT examination, lytic infiltrative lesions were observed extending from the scapula corpus to the glenoid and coracoid process and completely infiltrating the glenoid and sclerotic lesions in the part of the humerus head facing the glenoid (Figure 2). MRI examination could not be performed as the patient had a cardiac pacemaker implanted. In the PET-CT examination, as increased FDG accumulation was observed in the right scapula corpus and glenoid process (Figure 3), lesion excision with Tikhoff-Linberg type 4 resection was performed (Figure 4). As there was metastatic involvement in the proximal humerus, it was resected and fixed to the distal clavicle using an aortic graft (Figure 5).

## Discussion

Shoulder lesions can present with pain and palpable swelling detected incidentally on radiographs or pathological fractures. A detailed family history must be taken to determine the duration and character of the pain, symptoms must be evaluated, and, following a physical examination, radiographic examinations should be performed. The location of the patient's bone or soft tissue lesion, their age, and their radiographic characteristics all serve as guiding factors in the diagnosis [9].

The literature has frequently described malignant lesions of the scapula. In a study of 194 cases, Cleeman et al. stated that 21% of all scapular lesions were metastatic [10]. Samilson et al. reported that of 271 scapular tumors, 79 (29%) were benign, 168 (62%) were primary malignant tumors, and 24 (9%) were metastatic [11]. In the current study of 202 patients, 105 lesions (51.98%) were evaluated as benign tumors of which 81 were benign bone lesions and 24 were benign soft tissue lesions. Of the cases determined as benign bone tumors, 40 (37.03%) were evaluated as cystic lesions. According to the literature, simple bone cysts are the most common lesions around the shoulder and are more common in men, with a male-to-female ratio of 2-3:1. These lesions occur in the second decade of life in approximately 80% of patients, are generally solitary, and are located in long bones. Localization is in the proximal humerus, followed by the proximal femur in 80% of cases [12]. The most common benign tumors in the skeleton are enchondromas. Enchondromas are mostly seen in the tubular bones of the hand. In long bones, enchondromas frequently affect the femur, humerus, and tibia [13]. Of the 81 patients in this study with benign bone lesions, 30 were diagnosed with cystic lesions, and 16 were diagnosed with enchondromas. The surgical treatment of benign bone tumors depends on clinical symptoms and anatomical location. If bone lesions do not exhibit scintigraphy development, pathological fracture risk, or malignant transformation, clinical observation should be carried out; surgical treatment is not necessary [14]. Curettage and bone grafting are the recommended treatments in cases of imminent fracture caused by a benign bone tumor.

Benign musculoskeletal lipomatous lesions are common in both soft tissue and bone. Fifty percent of all soft tissue tumors are soft tissue lipomas. Radiological evaluation is diagnostic in 71% of cases [15]. Many benign soft tissue tumors are around the shoulder (6.3%), of which 64% are lipomas [16] Of the 24 patients with benign soft tissue tumors in the current study, 10 (41.66%) were diagnosed with lipoma. They were treated surgically in case of chronic pain.

The skeletal system is widely involved in advanced-stage cancer cases. Bone metastases can be classified as osteolytic or osteoblastic depending on the predominance of lysis or sclerosis in the bone according to the characteristic radiographic appearance of the lesions [17]. The most commonly seen malignant lesions in the proximal humerus are metastatic tumors. The primary malignant bone lesions often seen in the proximal humerus are osteosarcoma, chondrosarcoma, and Ewing sarcoma. Osteosarcoma is the most commonly seen primary sarcoma of the bone, and the proximal humerus is the third most commonly involved region. Approximately 15% of all osteosarcomas involve the proximal humerus and 1-2% the scapula or clavicle [10]. Ewing’s sarcoma is the second most common malignant bone tumor during childhood and adolescence. Ewing’s sarcoma is commonly seen in the long bones; the diaphysis is the more typical location [18]. In this study, of the primary malignant bone tumors, 10 (37.03%) were diagnosed with Ewing sarcoma. Two (7.4%) were diagnosed with osteosarcoma.

Mesenchymal tumors are the most challenging diagnostic pathology. In 2020, the new WHO Classification of Tumours of Soft Tissue and Bone improved the standardization of the diagnosis of these tumors [19]. Soft tissue sarcomas are most common in large muscle groups of the extremity, chest wall, mediastinum, and retroperitoneum. It is most commonly located in the head and neck of children and most commonly in the extremities of adults [20]. In the current study of patients with malignant soft tissue tumors, seven (33.33%) were diagnosed as malignant mesenchymal tumors. The diagnosis also relies on an experienced pathologist familiar with malignant soft tissue tumors.

Following the lungs and liver, the skeletal system is where metastatic carcinoma is most often seen. Any type of malignant tumor can metastasize to the bone. The most common malignancies are breast cancer in females and prostate cancer in males, but in recent years, there has been an increase in metastases secondary to lung cancer in both genders [21]. In the current study, 49 of the 97 malignant bone lesions were due to metastasis and were seen to be usually due to lung cancer. Bone metastases lead to pain, hypercalcemia, and complications such as medullar compression and pathological fracture. Long bone pathological fractures are the most severe complications which have high mortality rates [22]. According to a study by Saad et al., Pathological fractures have been linked to significantly lower survival rates for breast cancer, multiple myeloma, and prostate cancer [23]. Prophylactic surgical interventions, such as curative resection and reconstructive prostheses, lead to better survival rates in some patients than osteosynthesis of pathological fractures. Other surgical procedures in long bone metastases are arthroplasty, intramedullary nailing, and bone cement injections [21].

This study has some limitations. Firstly, the retrospective design and relatively low number of patients did not allow statistical data to be obtained in some tumor subgroups. Secondly, the treatments were not given in detail; thus, there is a need for new studies to evaluate the treatment methods for tumoral lesions around the shoulder. However, the numbers of benign and malignant tumors were almost equal. After all, the patients included in this study were only those who sought evaluation from the Council because simple lesions typically require outpatient polyclinic follow-up and do not necessitate a multidisciplinary approach or review by the Council.

## Conclusions

The shoulder girdle possesses a complex anatomical structure and ranks as the third most frequent site for primary tumors. Among the prevailing conditions are degenerative joint disease, rotator cuff pathologies, chronic infections, and secondary changes resulting from trauma. It is important to be mindful of benign or malignant tumoral pathologies in elderly patients who are rapidly developing massive masses and non-traumatic fractures. It is important to keep in mind that lytic lesions near the shoulder may be lung-origin metastases because of their close proximity, especially in elderly patients.
